# Supplementary material for: The interactome of intact mitochondria by cross-linking mass spectrometry provides evidence for coexisting respiratory supercomplexes
Source: Mol Cell Proteomics. 2017 Dec 8;17(2):216–32. doi: 10.1074/mcp.RA117.000470 (PMC5795388; doi:10.1074/mcp.RA117.000470)
Supplement: Supplemental Data [file supp_RA117.000470_133922_0_supp_23978_fzffwf.zip › spectra_annotation/mito_spectra_annotation/368-1-4-1-9-1.pdf]

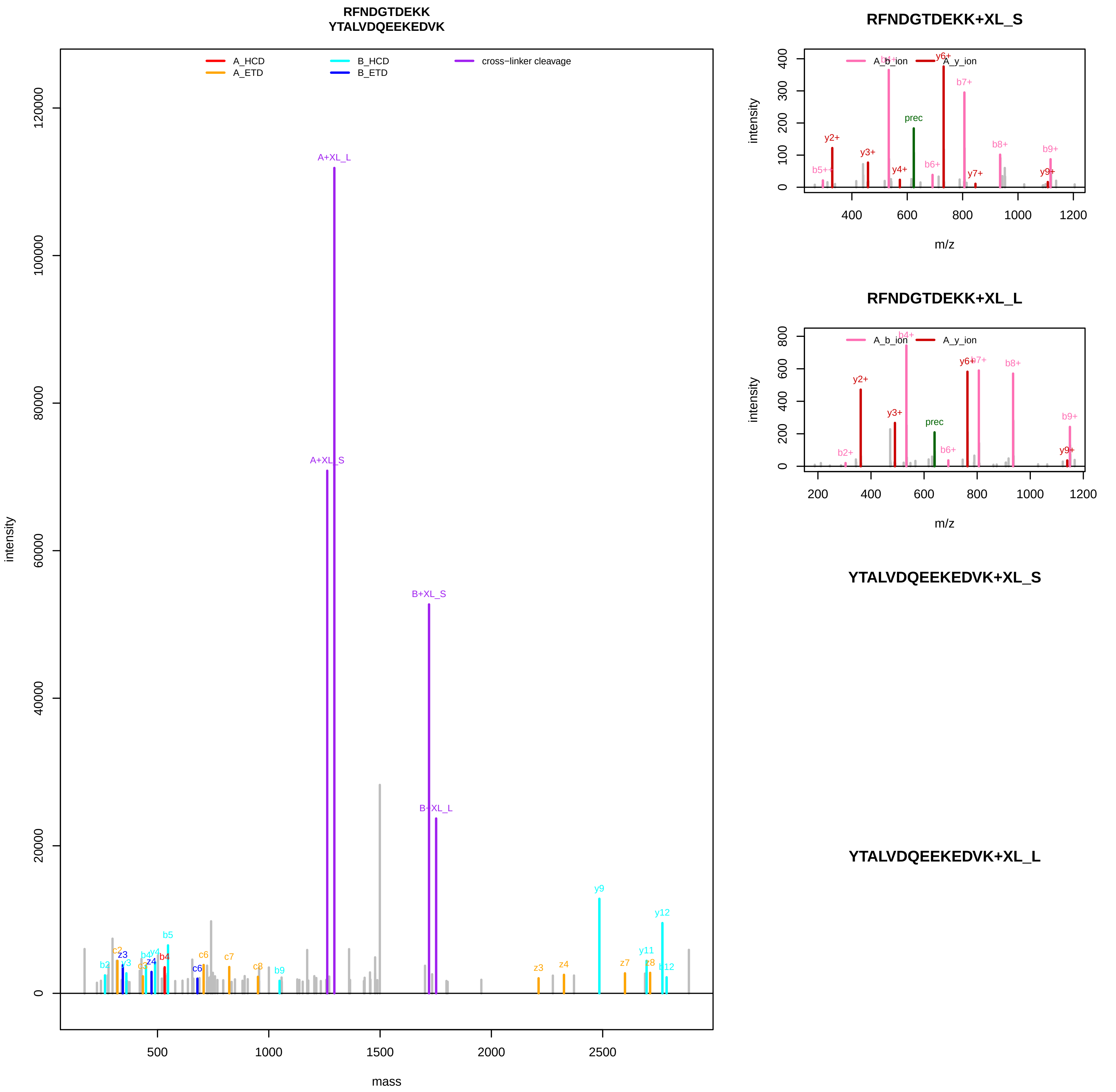

RFNDGTDEKK
YTALVDQEEKEDVK
A_HCD
A_ETD
B_HCD
B_ETD
cross−linker cleavage
A+XL_L
A+XL_S
B+XL_S
B+XL_L
b2
c2
z3
y3
c3
b4
y4
z4
b4
b5
c6
c6
c7
c8
b9
z3
z4
y9
z7
y11
z8
y12
b12
intensity
mass
0
20000
40000
60000
80000
100000
120000
500
1000
1500
2000
2500
RFNDGTDEKK+XL_S
A_b_ion
A_y_ion
y6+
b7+
prec
y2+
y3+
y4+
b6+
y7+
b8+
b9+
b5++
y9+
intensity
m/z
0
100
200
300
400
400
600
800
1000
1200
RFNDGTDEKK+XL_L
A_b_ion
A_y_ion
b4+
y2+
y3+
prec
y6+
b7+
b8+
b9+
b2+
b6+
y9+
intensity
m/z
0
200
400
600
800
200
400
600
800
1000
1200
YTALVDQEEKEDVK+XL_S
YTALVDQEEKEDVK+XL_L
